# Electrophysiological evaluation of incidental superior vena cava isolation following pulsed field ablation: a case report

**DOI:** 10.1093/ehjcr/ytag027

**Published:** 2026-01-23

**Authors:** Shin Hasegawa, Taiji Miyake, Yoriaki Shinoda, Mutsumi Aoyama, Hitoshi Matsuo

**Affiliations:** Department of Cardiology, Gifu Heart Center, 4-14-4 Yabutaminami, Gifu City, Gifu, 500-8384, Japan; Department of Cardiology, Gifu Heart Center, 4-14-4 Yabutaminami, Gifu City, Gifu, 500-8384, Japan; Department of Clinical Engineering, Gifu Heart Center, 4-14-4 Yabutaminami, Gifu City, Gifu, 500-8384, Japan; Department of Clinical Engineering, Gifu Heart Center, 4-14-4 Yabutaminami, Gifu City, Gifu, 500-8384, Japan; Department of Cardiology, Gifu Heart Center, 4-14-4 Yabutaminami, Gifu City, Gifu, 500-8384, Japan

**Keywords:** Atrial fibrillation, Case report, Dormant conduction, Pulsed field ablation, Pulmonary vein isolation, Unintended ablation

## Abstract

**Background:**

Pulsed field ablation (PFA) is increasingly adopted for atrial fibrillation (AF) due to its efficacy and lower risk of complications. However, uniform transmural energy delivery may induce excessive tissue modification and unintended effects on adjacent structures. Pulsed field ablation of the right superior pulmonary vein (RSPV) can result in conduction delay within the superior vena cava (SVC), but its electrophysiological properties remain poorly characterized.

**Case summary:**

An 81-year-old man with ischaemic cardiomyopathy, chronic heart failure, and paroxysmal AF underwent PFA for pulmonary vein isolation and posterior wall isolation. Pre-ablation mapping revealed myocardial sleeves within the SVC. Post-PFA mapping demonstrated complete SVC isolation. However, conduction recurred following adenosine triphosphate administration, and ectopic activity originated from the SVC. Circumferential radiofrequency ablation was subsequently performed, achieving durable SVC isolation.

**Discussion:**

This case demonstrates that PFA of the RSPV can lead to incidental SVC isolation. The presence of adenosine-induced dormant conduction highlights the potential for late reconnection, while the observation of ectopic activity supports the need for adjunctive ablation. Pulsed field ablation may unintentionally affect adjacent myocardial tissue, including the SVC and components of the cardiac conduction system. Therefore, careful electrophysiological assessment and monitoring of conduction recovery are essential to prevent arrhythmogenic triggers and to ensure durable isolation.

Learning points

- Pulsed field ablation of the right superior pulmonary vein can inadvertently cause superior vena cava (SVC) isolation.
- Adenosine-induced dormant conduction suggests the potential for late reconnection of unintended SVC isolation, whereas the proximity of the injury site to the sinus node poses a risk of sinus node dysfunction.
- Careful electrophysiological assessment, monitoring for conduction recovery, and timely adjunctive radiofrequency ablation are essential to ensure durable isolation.

## Introduction

Pulsed field ablation (PFA) for atrial fibrillation (AF) has been rapidly gaining clinical adoption due to its non-inferiority in outcomes compared to thermal ablation, with a lower incidence of complications such as oesophageal injury and pulmonary vein (PV) stenosis.^1^ However, the homogeneous transmural energy delivery of PFA may lead to excessive tissue modification.^2^ In addition, unintended energy delivery to adjacent structures has been reported.^2–5^

Application of PFA to the right superior pulmonary vein (RSPV) induces conduction delay in the superior vena cava (SVC), and while anatomical features and high-density mapping findings have been reported, electrophysiological characteristics remain poorly described.^4,6^

We present a case in which sinus rhythm mapping of the right atrium (RA) and SVC was performed before and after PFA-based pulmonary vein isolation (PVI) and left atrial posterior wall isolation (PWI), resulting in incidental SVC isolation. Based on evidence of potential reconnection, additional radiofrequency ablation (RFA) was performed.

## Summary figure

**Figure:**
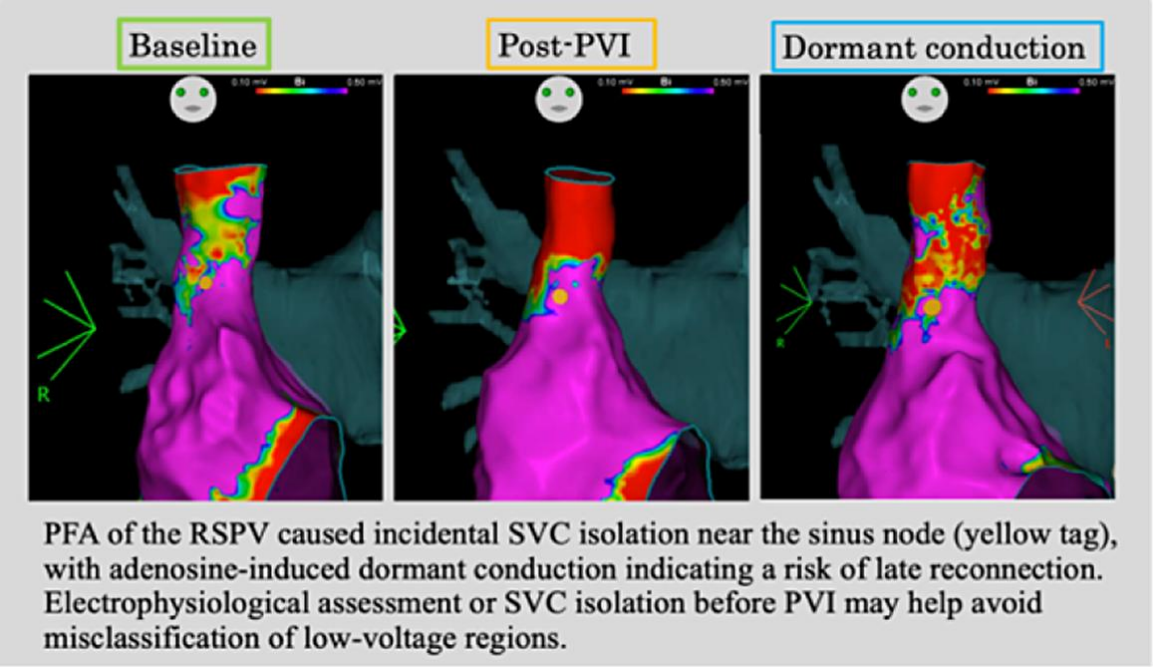


## Case presentation

An 81-year-old man with ischaemic cardiomyopathy after coronary artery bypass grafting, chronic heart failure, and a prior cerebral infarction presented to our hospital complaining of dyspnoea. He was diagnosed with congestive heart failure, with coarse crackles noted on examination, and was commenced on optimal medical therapy. Transthoracic echocardiography demonstrated a left ventricular ejection fraction of 63.1% and a left atrial volume index of 29.3 mL/m^2^. Chest radiography revealed pulmonary oedema and pleural effusion, with a cardiothoracic ratio of 46.6%.

**Figure 1:**
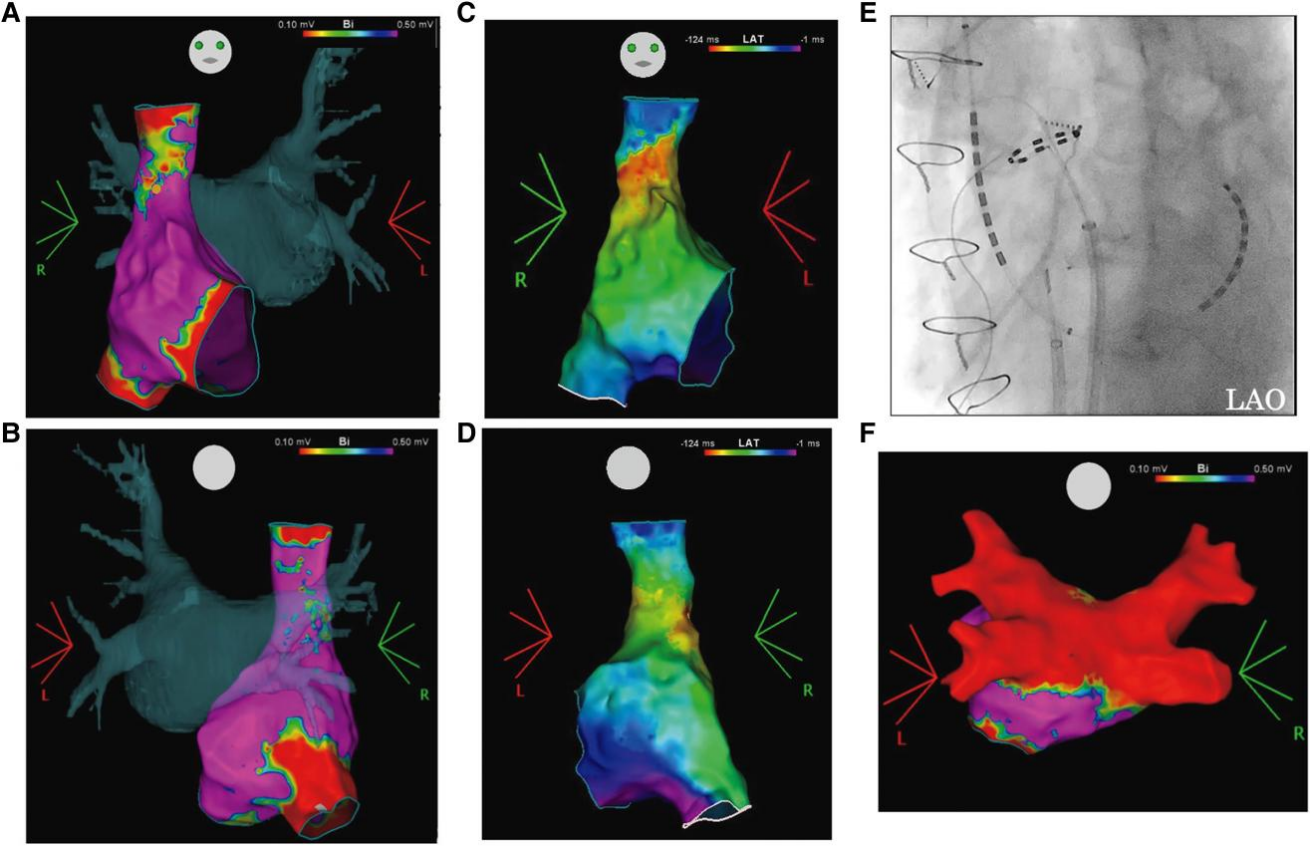
(*A* and *B*) Pre-ablation right atrial voltage map merged with left atrial computed tomography image. A myocardial sleeve extending from the superior vena cava was observed, reaching up to 35.6 mm from the superior border of the sinus node (yellow tag). (*C* and *D*) Pre-ablation right atrial activation map merged with left atrial computed tomography image. (*E*) Fluoroscopic images of pulsed field ablation (PFA) of the right superior pulmonary vein using a PulseSelect catheter. (*F*) Post-pulsed field ablation left atrial voltage map showing low voltage in the pulmonary veins and posterior wall.

Despite medical treatment, the patient’s heart failure worsened due to drug-refractory paroxysmal AF. After obtaining informed consent, a rhythm-control strategy with catheter ablation, including PVI and PWI, was planned. The procedure was performed under conscious sedation. Heparin was administered to maintain an activated clotting time of 350–400 s. A 20-electrode catheter (BeeAT; Japan Lifeline, Tokyo, Japan) was placed in the coronary sinus. Three-dimensional electroanatomical mapping (CARTO; Biosense Webster, Diamond Bar, CA, USA) of the RA and LA was performed during sinus rhythm using an Octaray mapping catheter (Biosense Webster, Diamond Bar, CA, USA). Myocardial sleeves were observed circumferentially in the SVC, extending up to 35.6 mm above the sinus node (*Figure 1A–D*). After confirming the anatomical location using intracardiac echocardiography and fluoroscopy, a single transseptal puncture was performed with a 10 Fr FlexCath Contour sheath (Medtronic, Minneapolis, MN, USA). Pulsed field ablation was delivered using the PulseSelect PFA system (Medtronic, Minneapolis, MN, USA) (*Figure 1D*). Pulmonary vein isolation was performed with eight applications to each PV. Posterior wall isolation was conducted with wires anchored in the LSPV and LIPV, delivering eight applications each to the roof and bottom lines. Post-ablation LA voltage mapping confirmed complete isolation of the PVs and posterior wall (*Figure 1E*).

**Figure 2:**
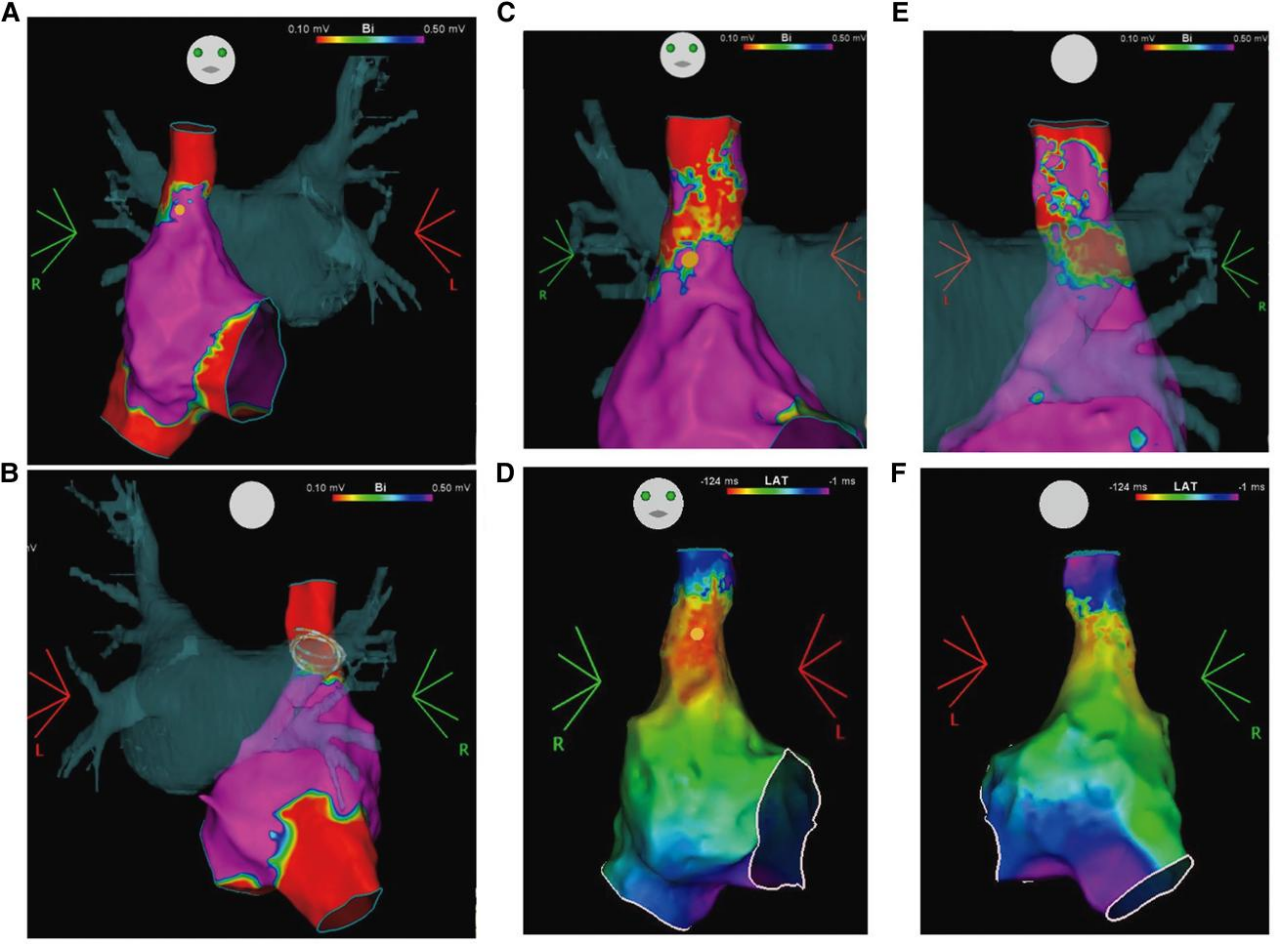
(*A* and *B*) Post-pulmonary vein isolation right atrial voltage map merged with left atrial computed tomography image. A low-voltage area was identified 5.8 mm from the superior border of the sinus node (yellow tag). Rings indicate pulsed field ablation application sites in the right superior pulmonary vein. (*C* and *D*) Right atrial voltage maps after pulmonary vein isolation, merged with left atrial computed tomography image, demonstrating dormant conduction. (*E* and *F*) Right atrial activation maps after pulmonary vein isolation, merged with left atrial computed tomography image, also demonstrating dormant conduction. The shortest distance between the sinus node (yellow tag) and the injured superior vena cava was 4.1 mm.

**Figure 3:**
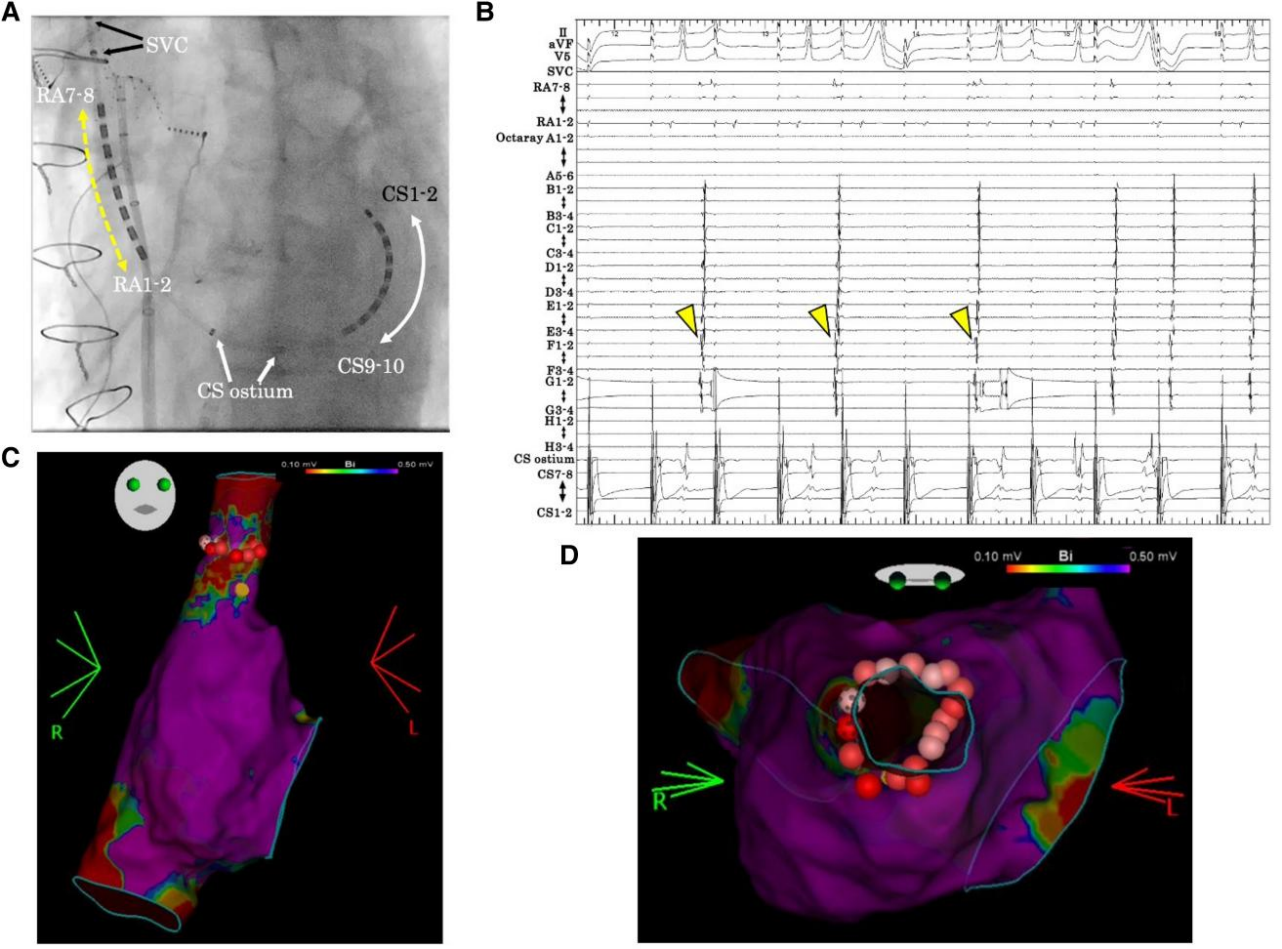
(*A*) Fluoroscopic images showing the position of an A BeeAT catheter in the superior vena cava (black arrow), right atrium (yellow arrow), and coronary sinus (white arrow). The Octaray catheter is positioned in the superior vena cava. (*B*) Intracardiac electrograms after adenosine triphosphate administration. During constant pacing from coronary sinus 5–6, ectopic activity originating from the superior vena cava was observed (yellow arrow). (*C* and *D*) Right atrial activation map after pulmonary vein isolation, merged with left atrial computed tomography image, demonstrating dormant conduction. Superior vena cava isolation was performed using thermal ablation (red tag). High-power, short-duration applications (dotted red tags) were delivered selectively to avoid phrenic nerve injury. Circumferential lesions were created under guidance from three-dimensional electroanatomic mapping, which delineated the electrophysiological position of the sinus node.

Post-PVI RA mapping revealed circumferential low-voltage and complete isolation of the SVC (*Figure 2A* and *B*; Supplementary material online, *Figure S1*). Exit block was confirmed by constant pacing from the SVC (see Supplementary material online, *Figure S2*). However, conduction was restored following an intravenous injection of 10 mg adenosine triphosphate (ATP) (*Figure 2C–F*; Supplementary material online, *Figure S3*). Additionally, ectopic activity was observed in the SVC (*Figure 3A* and *B*). Owing to the possibility of late reconnection, circumferential RFA was performed (*Figure 3C* and *D*). Ablation was performed using a QDOT Micro catheter (Biosense Webster, Diamond Bar, CA, USA) at 30 W, targeting an ablation index of 350. The phrenic nerve location was assessed by pacing, and diaphragmatic motion was continuously monitored under fluoroscopy. Bidirectional block and absence of dormant conduction were confirmed. Isoproterenol (ISP) was administered at 2 μg/min, and programmed stimulation failed to induce AF.

The post-operative heart rate was 76 b.p.m. No recurrence of AF was documented on 12-lead ECGs obtained during office visits over the 3-month follow-up period. The PQ interval was 158 ms, and neither symptomatic bradycardia nor sinus node dysfunction was observed. No evidence of phrenic nerve injury was detected on chest radiography.

## Discussion

The ADVENT trial demonstrated that PFA for AF is non-inferior to thermal ablation with respect to both efficacy and safety.^1,5–7^ Pulsed field ablation uses high-voltage, short-duration electrical pulses to create irreversible pores in cell membranes, leading to cell death and subsequent fibrosis through a non-thermal mechanism.^4^ Because of its myocardial selectivity, PFA is expected to provide safety advantages and achieve durable transmural lesions without injuring adjacent structures such as the oesophagus.^8^ However, excessive and incidental tissue modification remains a concern.^9^ Pulmonary vein isolation may predispose to macro-reentrant atrial tachycardia by creating critical isthmuses on the posterior wall^5^; therefore, we performed PWI to reduce this risk. Moreover, unintended isolation of the left atrial appendage (LAA) has been reported in cases with a narrow anteroposterior diameter of the LSPV and parallel alignment of the LSPV and LAA orifices.^3^ In addition, PFA of the LA may also affect the SVC. Matsuura *et al*. reported that new low-voltage areas (<0.5 mV, area ≥0.5 cm^2^) in the SVC were observed in 82.4% of patients after PFA, with 14.7% exhibiting circumferential involvement—occurring more frequently than after thermal ablation.^4,10^

The RSPV–SVC proximity and SVC deformation due to adjacent structures have been associated with SVC conduction delay after PFA.^4,10^ In our case, the shortest distance between the RSPV and SVC was 3.5 mm, and the SVC maintained a near-circular configuration on CT. Pulsed field ablation within the RSPV resulted in extensive low-voltage areas in the SVC, suggesting unintended energy delivery.

We evaluated SVC conduction properties following incidental isolation. Dormant conduction after thermal ablation has been associated with late reconnection.^11^ Weyand *et al*. reported dormant conduction in 6.7% of patients undergoing PFA following adenosine challenge, which was successfully eliminated by additional ablation.^11^ A case report demonstrated reconduction after incidental SVC isolation following PFA upon ISP administration; however, adenosine-induced dormant conduction after incidental SVC isolation with PFA has not been previously reported.^12^ In our case, the posterior-septal SVC adjacent to the RSPV and part of the anterior wall remained low voltage, whereas reconduction was observed in the other regions.

This observation suggests transient conduction impairment with potential for late recovery,^4^ while lesion depth during PFA—dependent on the degree and distance of catheter–tissue contact^13^—could have contributed to incomplete lesion formation between the RSPV and SVC, increasing the risk of late reconnection. Moreover, the SVC exhibited ectopic activity, raising concern for potential AF triggers and thereby justifying adjunctive RFA. Subsequent ATP administration confirmed the absence of dormant conduction.

The minimum distance between the sinus node and low-voltage SVC region was 4.1 mm. Sinus node dysfunction has been reported following SVC isolation,^14^ and transient conduction system injury after PFA has also been documented.^6,15^ The findings highlight the importance of careful consideration of accidental SVC modification and the risk of sinus node injury, particularly in patients with pre-existing sick sinus syndrome. Histological studies have shown that myocardial sleeves extend from the RA into the SVC, frequently exhibiting discontinuities.^16^ In the present case, isolation of the SVC near the sinus node was necessary. As the risk of sinus node injury could not be entirely excluded with PFA, RFCA was selected to safely perform the isolation.^17^ Circumferential lesions were created after confirming the position of the sinus node.

Unintended isolation in such cases may result in unnecessary ablation, suggesting that pre-procedural evaluation of SVC anatomy and electrophysiology is advisable prior to PVI.

In our case, PFA performed at the RSPV induced unintended SVC isolation, with adenosine administration revealing dormant conduction that required additional energy delivery. These findings indicate that PFA has the potential to create extensive lesions that may inadvertently involve adjacent myocardial structures, underscoring the need for careful procedural planning and caution in clinical practice.

This report is limited by uncertainty about long-term reconnection rates and the need for re-intervention after incidental SVC conduction delay following RSPV–PFA. Additional studies are needed to elucidate the long-term impact of PFA on adjacent cardiac structures.

## Lead author biography


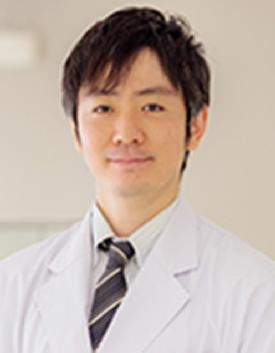


Shin Hasegawa graduated from Gifu University School of Medicine in 2015 and completed his initial training there until 2017. Since 2017, he has been working in the Department of Cardiology at Nagoya Tokushukai General Hospital. In 2025, he began working at Gifu Heart Center. His clinical and research interests within cardiovascular medicine include the management of structural heart disease, arrhythmias, and aortic diseases.

## Supplementary Material

ytag027_Supplementary_Data

## Data Availability

The data that support the findings of this study are available from the corresponding author upon reasonable request. No restricted data were used in this study.
