# Supplementary figures and images for: Electrophysiological evaluation of incidental superior vena cava isolation following pulsed field ablation: a case report
Source: Eur Heart J Case Rep. 2026 Jan 23;10(2):ytag027. doi: 10.1093/ehjcr/ytag027 (PMC12903455; doi:10.1093/ehjcr/ytag027)

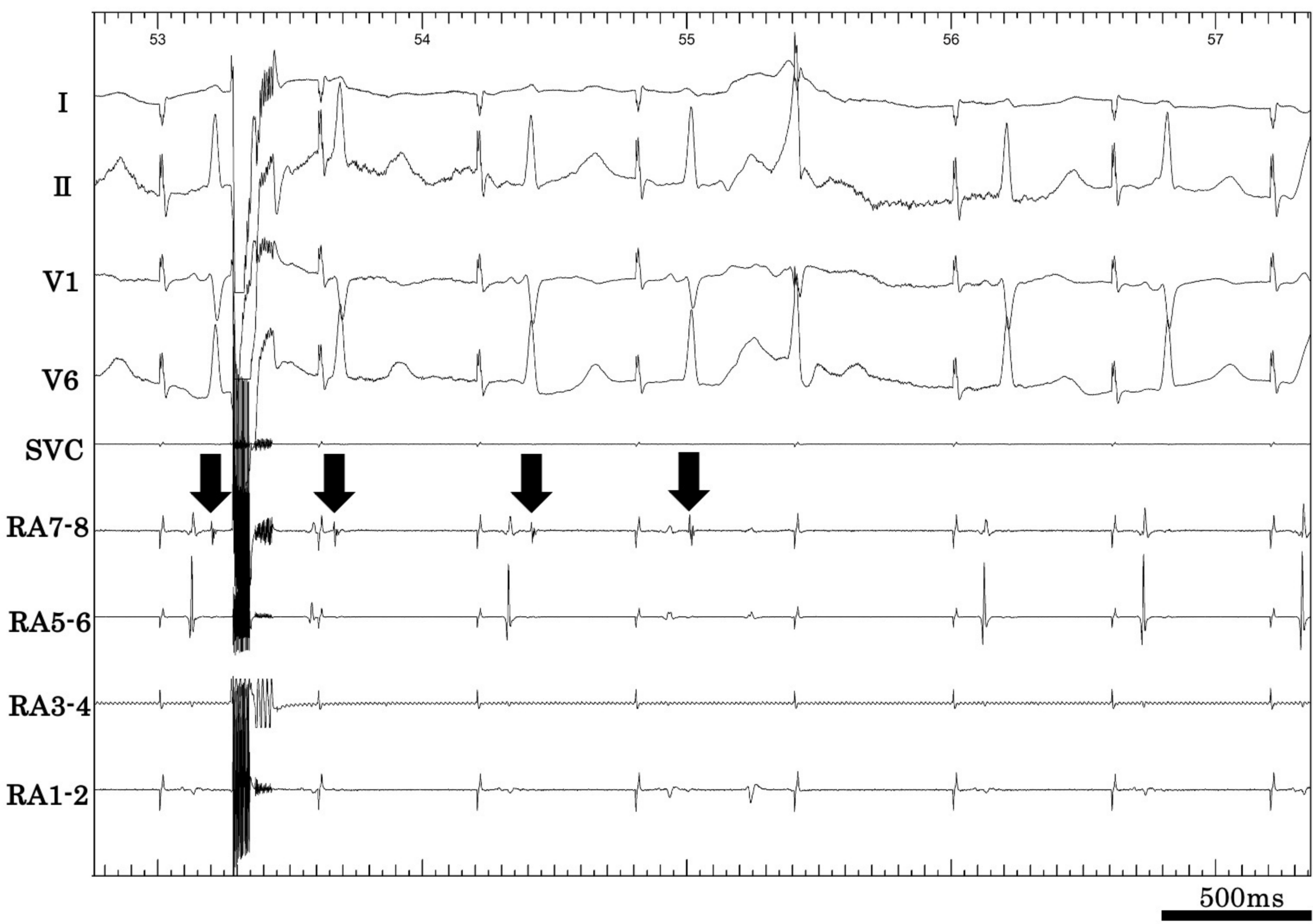

Supplement: ytag027_Supplementary_Data [file ytag027_supplementary_data.zip › Supplemental figure 1.pdf]

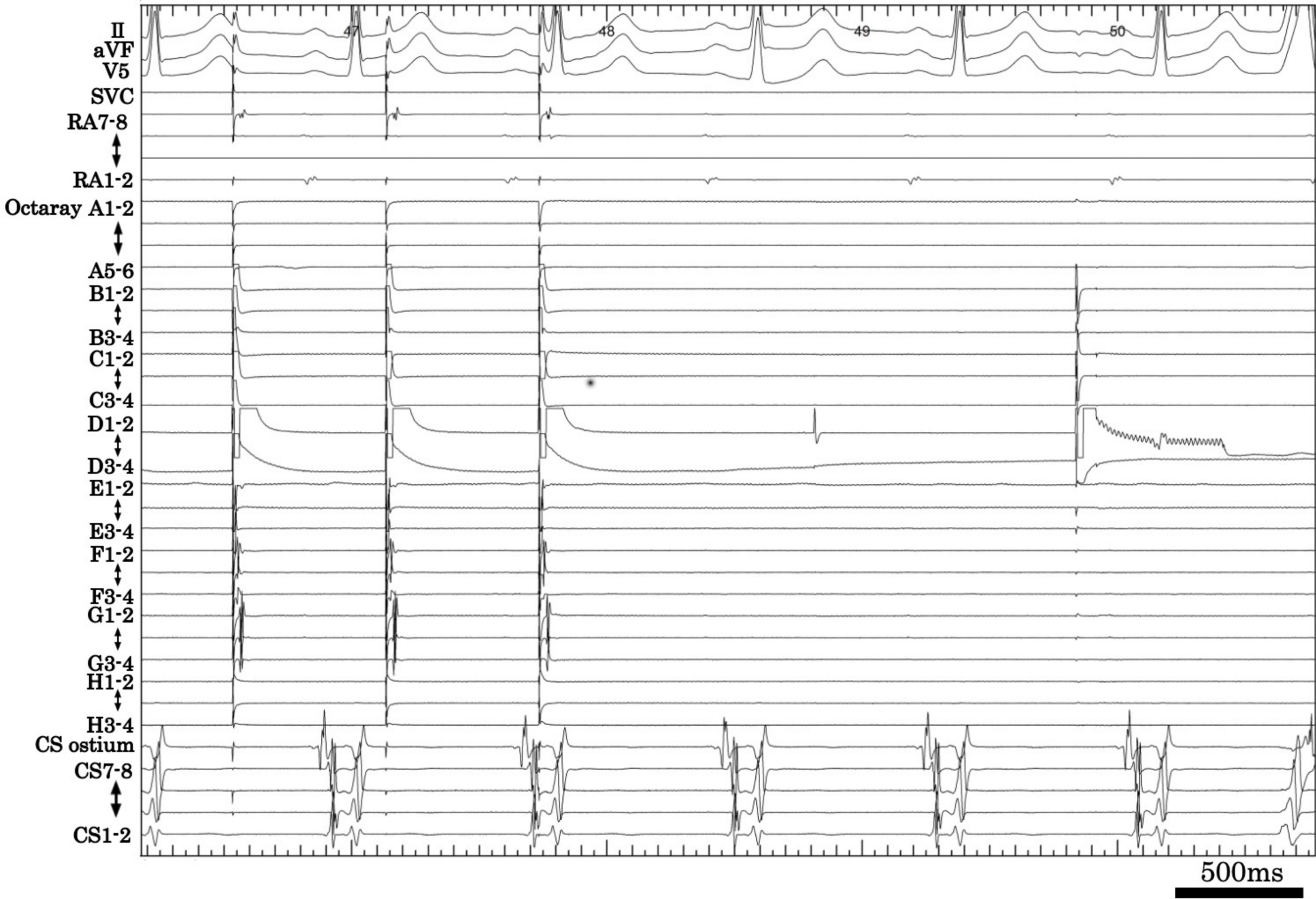

Supplement: ytag027_Supplementary_Data [file ytag027_supplementary_data.zip › Supplemental figure 2.pdf]

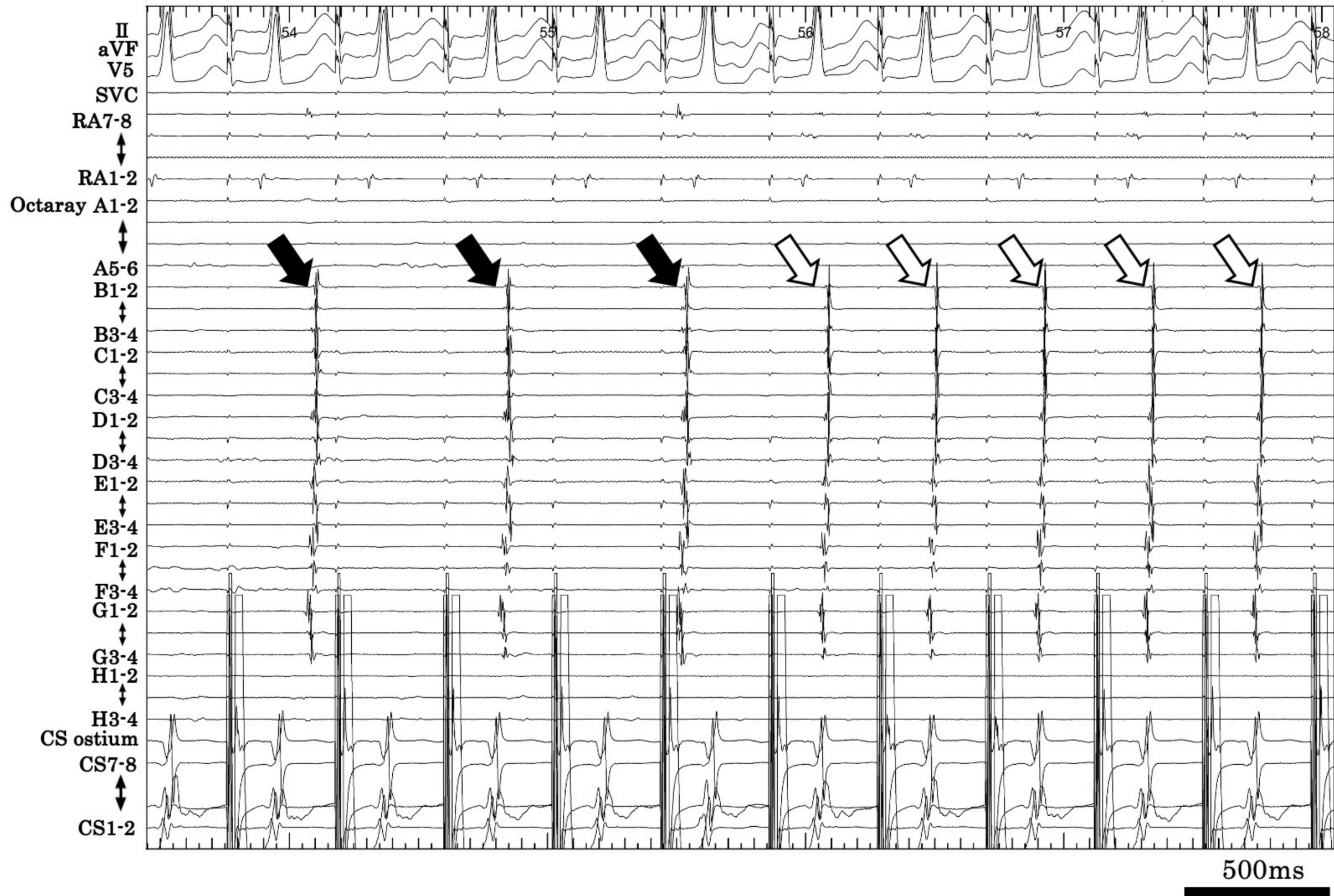

Supplement: ytag027_Supplementary_Data [file ytag027_supplementary_data.zip › Supplemental figure 3.pdf]
